# Evaluation of diversity among common beans (*Phaseolus vulgaris L*.) from two centers of domestication using 'omics' technologies

**DOI:** 10.1186/1471-2164-11-686

**Published:** 2010-12-02

**Authors:** Meghan M Mensack, Vanessa K Fitzgerald, Elizabeth P Ryan, Matthew R Lewis, Henry J Thompson, Mark A Brick

**Affiliations:** 1 Cancer Prevention Laboratory, Department of Horticulture, Colorado State Univ., Fort Collins, CO, 80523-1173, USA; 2 Department of Clinical Sciences, College of Veterinary Medicine and Biomedical Sciences, Colorado State Univ., Fort Collins, CO, 80523-1678, USA; 3 Proteomics and Metabolomics Facility, Department of the Vice President for Research, Colorado State Univ., Fort Collins, CO, 80523-2021, USA; 4 Department of Soil and Crop Sciences, Colorado State Univ., Fort Collins, CO, 80523-1173, USA

## Abstract

**Background:**

Genetic diversity among wild accessions and cultivars of common bean (*Phaseolus vulgaris *L.) has been characterized using plant morphology, seed protein allozymes, random amplified polymorphic DNA, restriction fragment length polymorphisms, DNA sequence analysis, chloroplast DNA, and microsatellite markers. Yet, little is known about whether these traits, which distinguish among genetically distinct types of common bean, can be evaluated using omics technologies.

**Results:**

Three 'omics' approaches: transcriptomics, proteomics, and metabolomics were used to qualitatively evaluate the diversity of common bean from two Centers of Domestication (COD). All three approaches were able to classify common bean according to their COD using unsupervised analyses; these findings are consistent with the hypothesis that differences exist in gene transcription, protein expression, and synthesis and metabolism of small molecules among common bean cultivars representative of different COD. Metabolomic analyses of multiple cultivars within two common bean gene pools revealed cultivar differences in small molecules that were of sufficient magnitude to allow identification of unique cultivar fingerprints.

**Conclusions:**

Given the high-throughput and low cost of each of these 'omics' platforms, significant opportunities exist for their use in the rapid identification of traits of agronomic and nutritional importance as well as to characterize genetic diversity.

## Background

Common bean (*Phaseolus vulgaris *L.) is one of the oldest cultivated crops in the Americas and is the most important grain legume for human consumption with production more than double that of the second most important grain legume, chickpea (*Cicer arietinum *L.) [1]. Common bean was domesticated in the Americas by indigenous people during pre-Colombian times. Archeological data suggest that bean was independently domesticated in different regions of the Americas including, the Andean region of South America [2], Argentina [3], and Mexico [4,5]. The oldest domesticated beans were found at archeological sites in each of these regions between 4300 and 8000 B.P. [6,7] Changes in bean plant phenotype as a result of domestication include but are not limited to growth habit, seed size, seed retention, and maturity[3,8]. However, the molecular events that underlie these differences in agronomic traits have not been elucidated.

**Figure 1:**
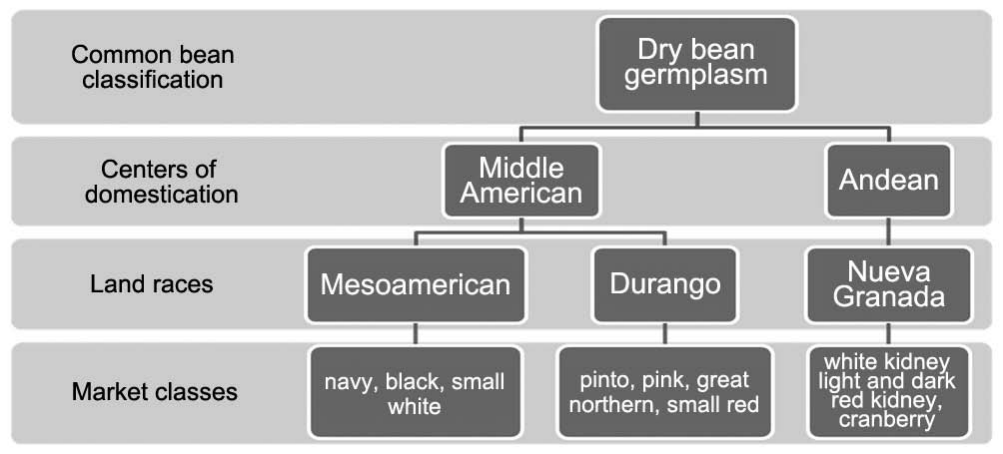
**Phylogenetic relationship between center of domestication (COD) and races used in the study**.

Original classification of common bean germplasm was performed by Singh et al. [9] into two primary Centers of Domestication (COD); namely Middle American from Central and North America and Andean from South America. Primary CODs were further divided into Races based on geographic origin and genetic lineage. The Andean COD was subdivided into three Races: Nueva Granada (Columbia), Peruvian (Peruvian highlands), and Chilean (northern Chile and Argentina). The commercial market classes that represent Race Nueva Granada in the USA, include light red kidney, dark red kidney, white kidney, and cranberry beans. Andean beans such as Calima, Azufrado, sugar bean, and other mottled types are also widely grown in Africa and the Caribbean. Beans from the Middle American COD were domesticated in West-Central Mexico [10] and include Durango (central highlands of Mexico), Jalisco (coastal Mexico near the state of Jalisco), and Mesoamerican (lowland tropical Central America) Races. Market classes grown in the US that typify Race Durango include pinto, great northern, small red, and pink bean. Navy, small white, and black beans represent the Mesoamerica landrace. The organization of common bean into COD and Races is depicted in Figure 1.

Current tools used to characterize genetic diversity within common bean include plant morphology [11], seed protein allozymes [11-14], random amplified polymorphic DNA[15], restriction fragment length polymorphisms [16], DNA sequence analysis [17], chloroplast DNA [18], and microsatellite markers [19,20]. While effective for evaluating genetic diversity of parent plants, these techniques are of limited use in breeding programs focused on crop improvement. Methods that can assess potential differences in biological function among cultivars on a genome wide basis have become available in high-throughput and low-cost formats. These 'omics' platforms provide an unprecedented opportunity to ultimately identify the underlying molecular mechanisms that account for traits of agronomic and nutritional importance.

The research presented herein utilized three 'omics' platforms (transcriptomics, proteomics, and metabolomics) to determine whether beans from Middle American and Andean COD differ in their patterns of gene transcription, protein expression, and/or small molecule synthesis and metabolism. To our knowledge, this is the first report that shows differences in gene transcription, protein expression, and metabolite profiles between beans from two COD using 'omics' techniques. Moreover the metabolomic approach was used to evaluate whether common bean cultivars within the same gene pool, referred to as a market class, differ in their profile of metabolites. These proof-in-principle experiments herald significant opportunities for utilizing 'omic' technologies in the rapid identification of traits with agronomic and nutritional importance that could serve to guide common bean breeding programs.

## Results

### Three 'omics' platforms to distinguish common bean COD

Experiments were conducted to determine whether or not the three 'omics' technology platforms (transcriptomics, proteomics, and metabolomics) would be capable of distinguishing dry bean cultivars from each of the two COD. The approach used was sequential; transcriptomics experiments were performed first, followed by proteomics, and then metabolomics, with increasing complexity of the experimental design as work progressed to each new platform. The white kidney bean and navy bean market classes representing the Andean and Middle American gene pools were selected as representative of the respective COD. Furthermore, both market classes are commercially important, and had a white pigmented seed coat thus limiting the likelihood of identifying qualitative differences between COD that were due solely to synthesis of different pigments.

### Transcript profiling

**Figure 2:**
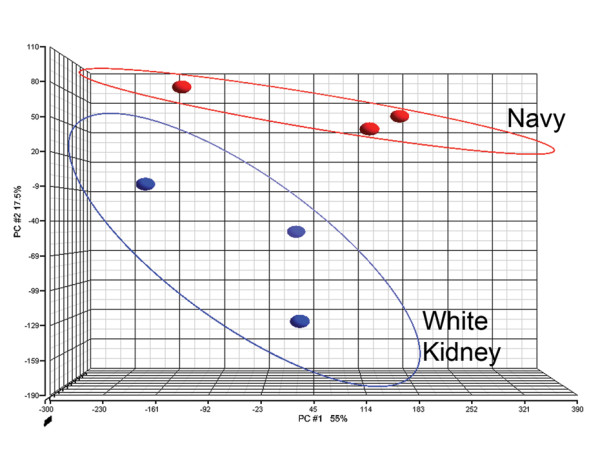
**Transcriptomic analysis of navy bean and white kidney bean derived from microarray analysis of gene expression**. Principle components analysis of navy (red) and white kidney (blue) harvested 2 wk after plant flowering. Three biological replicates of bean cDNA were hybridized to spotted soybean microarrays.

Developing seeds from the Middle American COD navy bean cultivar Norstar and the Andean COD white kidney bean cultivar Silver Cloud were evaluated for differential expression of transcripts. Initially an Affymetrix soybean microarray was used for this purpose. However, using the unmodified array, we found that the high binding specificity of Affymetrix microarrays, resulted in a hybridization efficiency of *P. vulgaris *cDNA onto the soybean microarray (< 10%) that was too low for expression analysis. Although it has been recently reported that the use of masking biased probes overcomes this limitation [21], a spotted cDNA microarray for soybean was selected as an alternative approach because of their successful use in cross-species studies [22]. This cDNA soybean microarray, available through the Keck Center for Comparative and Functional Genomics at the University of Illinois, has been previously described [23]. Principal component (PC) analysis showed that navy and white kidney gene expression profiles differed when seeds were collected 2 wk after anthesis (Figure 2). The clones represented on the microarray were previously annotated as described by Vodkin et al. [23] and supplied with the microarray. Based upon that annotation, 330 clones were identified as differentially expressed between white kidney and navy at 2 wk after anthesis. The clones were selected by one-way ANOVA (p < 0.01) and a fold change cutoff in expression of 2.0. The majority of these clones (309) were up-regulated in white kidney bean and the remaining 21 were up-regulated in navy bean. Of these 330 differentially expressed clones, 30 were up-regulated in white kidney by at least 5-fold change and only 1 clone was up-regulated in navy by at least 5-fold change. The clones differentially expressed by 5-fold change were annotated and displayed in Table 1. The majority of clones up-regulated by 5-fold change in white kidney have a role in transcription, translation, and protein synthesis/modification based on the annotations provided with the microarray. Examples of up-regulated white kidney clones include: ribosomal proteins and ubiquitin. The only clone up-regulated in navy has homology to a ripening induced protein in 3 wk old seeds, there were 13 clones that were differentially expressed between the two beans by greater than 2-fold change (Table 2). Three clones were up-regulated in white kidney, 1 of which was annotated as nucleoside diphosphate kinase. Ten clones were up-regulated in navy bean, but only 4 were identified in the annotation and are involved in metabolism. The observation that fewer genes were expressed at 3 wk old compared to 2 wk old likely reflects the reduced metabolism in the developing seed as it approached physiological maturity.

**Table 1:** Phaseolus vulgaris genes differentially expressed between navy and white kidney bean seeds two weeks after flowering.

Clone ID	GenBank accession	Ratio WK:NB^a^	Annotation (BLAST hit and organism)	Function^b^
Gm-r1070-1164	AW570504	5.353	calmodulin 2 [Medicago truncatula]	cb
Gm-r1088-5593	BF067176	5.246	annexin [Medicago truncatula]	cs
Gm-r1083-4862	BE023117	5.143	peroxidase [Glycine max]	def
Gm-b10BB-41	AI495218	5.238	RUBISCO small chain 1 precursor [Glycine max]	en
Gm-r1089-1548	BU927150	5.667	nuM1 [Medicago sativa]	oth
Gm-r1021-3096	AI495362	7.943	ubiquitin [Glycine max]	pm
Gm-r1021-1452	AI441940	7.113	ubiquitin [Lycopersicon esculentum]	pm
Gm-r1070-6513	AW186354	6.617	pectin methylesterase-like protein [Arabidopsis thaliana]	pm
Gm-r1083-3778	BE020202	5.450	ubiquitin [Glycine max]	pm
Gm-r1083-4103	BE020700	5.362	pentameric polyubiquitin	pm
Gm-r1021-951	AI443187	5.352	polyubiquitin containing 7 ubiquitin monomers	pm
Gm-r1070-3321	AW423503	12.032	putative transcriptional coactivator [Brassica rapa]	txn
Gm-r1070-8054	AW508573	8.634	heat shock factor protein 3 (HSF3)/heat shock transcription factor 3 (HSTF3) [Arabidopsis thaliana]	txn
Gm-r1089-2093	BG352926	8.534	glutamyl-tRNA synthetase [Arabidopsis thaliana]	tln
Gm-r1021-1432	AI440898	7.871	translation initiation factor IF-1 [Glycine max]	tln
Gm-r1070-8367	AW508710	6.222	putative small nuclear ribonucleoprotein polypeptide E [Oryza sativa (japonica cultivar-group)]	tln
Gm-r1089-2264	BE330222	6.167	elongation factor 1-alpha, putative [Arabidopsis thaliana]	tln
Gm-r1070-7717	AW472122	9.702	ribosomal protein [Petunia × hybrida]	tln-rc
Gm-r1089-7404	BQ298634	6.190	40 S ribosomal protein S21 (RPS21C) [Arabidopsis thaliana]	tln-rc
Gm-r1070-4290	AW396000	6.157	putative ribosomal protein [Capsicum annuum]	tln-rc
Gm-r1088-5929	BG882122	5.877	putative protein [Arabidopsis thaliana]	tln-rc
Gm-r1021-1062	AI442633	5.829	60 S ribosomal protein L24 [Prunus avium]	tln-rc
Gm-r1088-5173	BG790456	5.397	40 s ribosomal protein S23 [Euphorbia esula]	tln-rc
Gm-r1021-74	AI444100	5.237	putative ribosomal protein S29 [Oryza sativa (japonica cultivar-group)]	tln-rc
Gm-r1088-5497	BF066304	5.140	ubiquitin fusion protein/40 S ribosomal protein S27a [Zea mays]	tln-rc
Gm-r1083-2977	AW703688	5.027	zinc finger (C3HC4-type RING finger) protein-related [Arabidopsis thaliana]	tln-rc
Gm-r1083-442	AW278239	11.708	unknown protein [Arabidopsis thaliana]	unk
Gm-r1089-5254	BG507608	7.211	expressed protein [Arabidopsis thaliana]	unk
Gm-r1070-8007	AW472347	6.549	farnesylated protein [Cicer arietinum]	unk
Gm-r1089-4543	BI701778	5.519	putative leucine-rich repeat protein [Arabidopsis thaliana]	unk
Gm-r1089-565	AW397679	5.474	LIM domain-containing protein [Arabidopsis thaliana]	unk
Gm-r1070-8492	AW508223	0.167^c^	ripening-induced protein [Fragaria vesca]	met

^a ^List of annotated genes differentially expressed (p < 0.01) by an average ratio > 5-fold change.

^b ^Function abbreviations: cb-calcium ion binding, cs-cellular structure, def-defense, en-energy, oth-other, pm-protein modification, txn-transcription, tln-translation, tln-rc-translation-ribosomal component, unk-unknown, met-metabolism.

^c ^Value under 0.2 indicates greater than 5-fold expression in navy bean compared to white kidney bean (written as white kidney:navy, WK:NB)

**Table 2:** Phaseolus vulgaris genes differentially expressed between navy and white kidney bean seeds three weeks after flowering.

Clone ID	GenBank accession	Ratio WK:NB^a, b^	Annotation (BLAST hit and organism)	Function
Gm-r1089-8863	BQ611197	3.241	nucleoside diphosphate kinase [Glycine max]	ATP binding
Gm-r1089-3127	CA937608	3.031	no match	unknown
Gm-r1070-9059	AW567598	2.755	no match	unknown
Gm-b10BB-31	AW707047	0.145	Dihydroxypterocarpan-6A-Hydroxy	secondary metabolism
Gm-r1089-6634	BM893235	0.167	nitrite transporter [Cucumis sativus]	transporter activity
Gm-r1070-5377	AW509221	0.147	oxygen evolving complex 33 kDa photosystem II protein [Nicotiana tabacum]	transferase activity
Gm-r1021-1612	AI441654	0.252	phototropic-responsive NPH3 family protein [Arabidopsis thaliana]	signal transducer activity
Gm-r1088-6395	BI785891	0.219	unknown [Arabidopsis thaliana]	unknown
Gm-r1070-8857	AW568996	0.346	unnamed protein product [Arabidopsis thaliana]	unknown
Gm-r1070-4383	AW100515	0.477	no match	unknown
Gm-r1070-3334	AW423614	0.344	no match	unknown
Gm-r1083-4041	BE020154	0.298	no match	unknown
Gm-r1070-7730	AW471962	0.260	no match	unknown

^a^List of annotated genes differentially expressed (p < 0.01) by an average ratio > 2-fold change.

^b^Value < 0.5 indicates > 2-fold expression in navy bean compared to white kidney bean (written as white kidney:navy, WK:NB).

### Proteomic profiling

Based on the transcript profiling data reported in the previous section, it was predicted that the profile of expressed protein would differ between cultivars representative of COD. Protein expression was assessed using a two-dimensional gel system instead of an LC-MS platform, so that a visual picture of similarities and differences in types and amounts of proteins expressed between COD could be obtained. Moreover, rather than limiting the analysis to one cultivar from each market class-COD category, three cultivars from each genepool were evaluated. The list of cultivars studied can be found in the Materials and Methods section. Master gels of the two dry bean germplasm (white kidney and navy) were created for purposes of exploratory analysis. Close visual inspection showed distinct differences in both the absolute number of proteins as well as differences in concentration (spot density) between COD. With the false discovery rate set at zero, 2186 spots were selected from the master gel using the PDQuest software.

**Figure 3:**
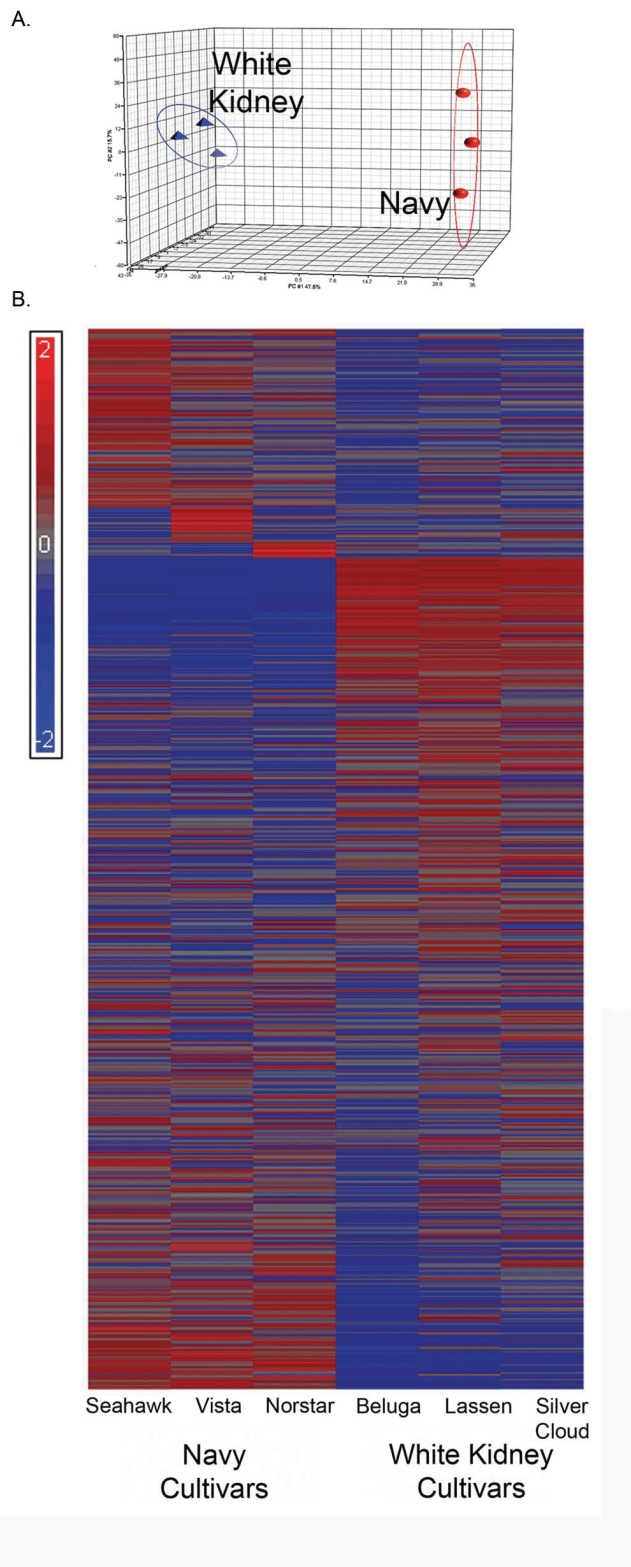
**Proteomic analysis of two dry bean market classes: white kidney and navy**. (a) PC analysis of three white kidney bean cultivars, "blue triangles" and three navy bean cultivars, "red circles". (b) Hierarchical clustering displayed with heat map of densitometry values from 2 D gels comparing three white kidney bean and three navy bean cultivars. Each cell in the heat map represents one protein. Mean spot density for each protein or protein fragment was calculated across all cultivars and intensities relative to the mean were plotted using a blue to red color map to indicate a decrease or increase in intensity with respect to the calculated mean spot density. Cultivars studied are listed in the Materials and Methods section.

Data from the three cultivars evaluated were combined and subjected to PC analysis (Figure 3a). A heat map of spot densities showed visual differences in up- and down-regulated proteins when comparing white kidney bean and navy bean germplasm (Figure 3b). One-way ANOVA analysis showed that 733 proteins were statistically different between the two COD (p < 0.05) with a fold change greater than 2. Of the 773 proteins, 282 proteins were up-regulated in white kidney bean and 254 proteins were up-regulated in navy bean.

### Metabolomic fingerprinting

**Figure 4:**
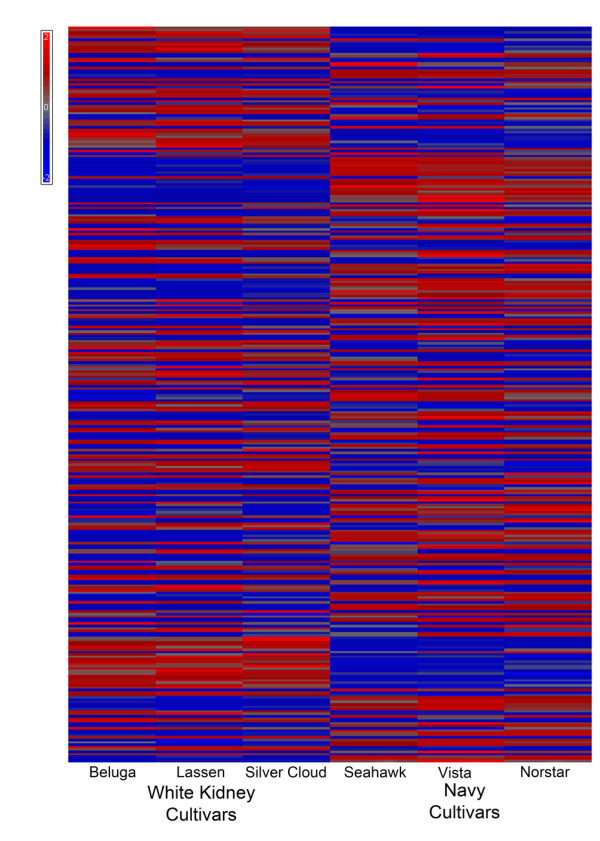
**Heat map generated using metabolomic analysis**. Each cell of the heat map represents one mass measurement and is colored to represent normalized fold change. Mean intensities for each mass were determined across all cultivars and intensities relative to the mean were plotted using a blue to red color map to indicate a decrease or increase in intensity with respect to the calculated mean intensities.

While the results of proteomic analyses were consistent with genetic differences between cultivars in each COD, these findings do not address the nature of these differences. It can be argued that the majority of the proteins detected participate in the synthesis, transformation, and degradation of both primary and secondary metabolites that play a role in plant architecture, reproduction, and defense against biotic and abiotic stresses [24]. The focus of the metabolomic analyses reported herein was on the small molecules having a mass less than 1000 Daltons. Our objective was to determine if these small molecule profiles differed between Andean and Middle American beans. The same market classes and cultivars evaluated in the proteomics experiments were metabolically fingerprinted. To perform unbiased metabolomic analyses, m/z values from 50-1000 were monitored using UPLC-ESI-MS revealing 6732 possible small molecules in positive mode. A heat map of differences in intensity of the small molecules between the cultivars from each COD is shown in Figure 4 where each column in Figure 4 corresponds to a specific cultivar. One-way ANOVA results showed that 472 small molecules corresponding to 7% of the total number of features detected were a minimum of 2 fold higher in Andean beans (p < 0.05); 487 small molecules, also approximately 7% of the total number of features detected, were 2 fold higher in Middle American beans (p < 0.05).

### Cultivated varieties distinguished by metabolomic fingerprinting

**Figure 5:**
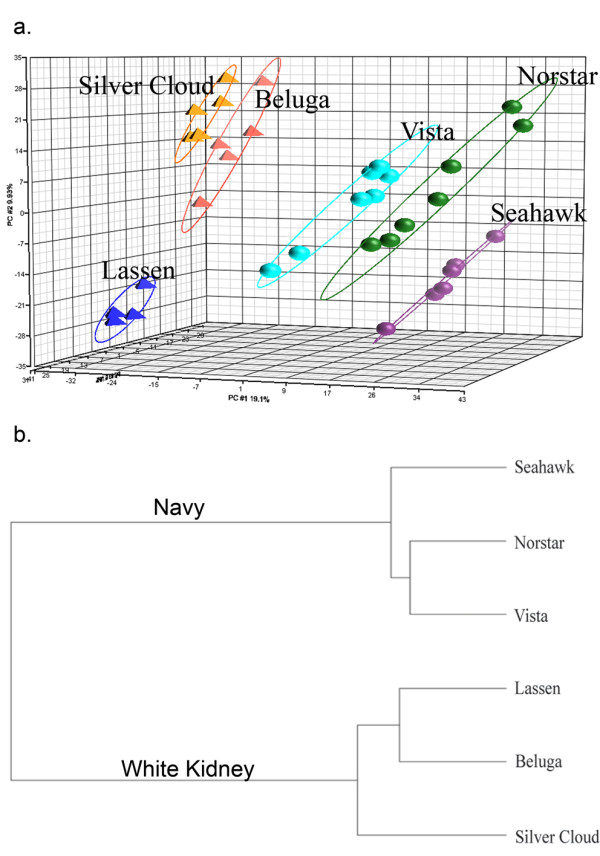
**Metabolomic analysis of bean cultivars within market classes**. (a) Normalized data shown as 3 D PC analysis of 6 different varieties of dry bean. Navy bean cultivars, "circles", included are Seahawk, Norstar, and Vista. White kidney cultivars, "triangles", included are Beluga, Silver Cloud, and Lassen. Bean material analyzed using UPLC/MS. 5-7 biological replicates per market class were extracted. Ellipses are drawn at 2 standard deviations from the mean for each market class. (b) Dendrogram showing relationship between six dry bean cultivars. Clustering determined using Euclidian distances.

Metabolomic fingerprinting was also utilized to assess differences in small molecule profiles among bean cultivars within market classes. Clear metabolite clustering of the cultivars within the two germplasms was observed using PC analysis and hierarchal clustering. As shown in Figure 5a, the bean cultivars were separated along PC 2 and PC 3, while PC 1 separated by COD. PC 1 explained 19.1% of the variation while PC2 and PC3 explained an additional 9.95% and 6.2% of the total variation among cultivars, respectively. Hierarchal clustering using Euclidian distances was used to distinguish among cultivars and between COD. The resulting dendrogram shows clustering of cultivars within COD (Figure 5b) which mimics clustering seen in the proteomics analysis. One-way ANOVA analysis of the 6 cultivated varieties grouped by market class showed 542 features were statistically different (p < 0.05) with a fold change of at least 2 between the navy bean and white kidney market classes. The 542 features were further analyzed to determine which could differentiate cultivars within the market classes. In this case, 167 features were found to be statistically different (p < 0.05) within the white kidney bean cultivars; whereas, 246 features were found to be statistically different (p < 0.05) between the three navy bean cultivars.

## Discussion

The elucidation of molecular differences underlying traits that permit the division of common bean germplasm into distinct centers of domestication has been largely unexplored by high-throughput 'omic' technologies. A triad of 'omic' technologies: transcriptomics, proteomics, and metabolomics, were used herein to determine whether differences in gene transcription, protein expression, or synthesis/metabolism of small molecules were of sufficient magnitude to distinguish among bean cultivars within and between two COD (Figure 1). As shown in Figure 2b, 3c, and 5a, distinct differences were observed among cultivars and between the two COD in all three 'omics' signatures.

The transcript profiling experiments revealed that chronological age of the seed from anthesis has a significant impact on the number of differentially expressed genes and the functions (gene ontology) associated with those genes. However, examination of the gene lists in Table 1 failed to provide a clear picture of the physiological distinctions between COD. One limitation to consider is that distinct *P. vulgaris *genes may exist without a soybean homologue to utilize during hybridization and thus a cross-species microarray may exclude unique dry bean transcripts. Reciprocally, the chance of false positives was increased in the cross-species use of the cDNA spotted arrays because of reduced hybridization stringency. The drawbacks associated with using a soybean array for common bean transcriptomics stem from reduced synteny between *P. vulgaris *and *G. max*. The most recent common ancestor for *P. vulgaris *and *G. max *was 19 Ma. At some time point following divergence, diploidization of the *G. max *genome occurred making its chromosome number 2n = 80 compared to 2n = 40 of *P. vulgaris *[25]. Genomic rearrangement including translocation or gene loss during diploidization of the soybean leads to reduced synteny between the two species and must be considered when attempting to determine chromosome overlap between the soybean and the common bean for genetic mapping. The use of the Affymetrix array platform as reported in Yang et al. [21] may serve to overcome some of the noted limitations, and it is worthwhile to note that the Whole-Genome Sequence of Common Bean project and the Ibero-American whole genome sequencing project include both COD [26]. With the entire common bean genome sequenced, a complete dry bean microarray can be synthesized and annotated to advance the transcriptomics platform Challenges in gene list interpretation also have been encountered using other organisms and have led to the development of alternative approaches to data analysis methods such as Gene Set Enrichment Analysis (GSEA) [27]. GSEA permits the investigator to use existing knowledge to test specific hypotheses about differences in pathways and networks of gene expression between two or more treatment conditions. Efforts to incorporate GSEA for market class stratification were limited by cross-species hybridization and the lack of dry bean and soybean gene lists. Consequently, no useful data was gained. This situation underscores the importance of current efforts to sequence the *Phaseolus *genome. When that work is completed, it will be possible to generate hypotheses for additional testing based on observations about plant morphology [11] or seed protein allozymes [11-13] that have been used to characterize genetic diversity within common bean. In addition, as work on metabolomic analyses continues and new compound libraries are constructed, metabolomic data such as that shown in Figure 4 and 5 will identify specific small molecules that distinguish cultivars within each COD and within a market class-COD grouping. That information will permit future hypothesis generation about the biosynthetic pathways that are induced or repressed to account for differences in metabolite profiles. Since differences in small molecule biosynthesis are likely to result not only from differences in gene transcription but also arise from differences in the translation of transcripts and post translational modifications of proteins, the potential contributions of proteomic analyses such as those shown in Figure 3 are clear. For future analyses, we recommend that an HPLC or UPLC separation approach be employed rather than 2DGE to interrogate the *Phaseolus *proteome because of the speed, throughput, and relative cost of the LC-based platform.

As one considers quantitative differences among the three 'omics' signatures that distinguished between the Andean and Middle American COD, it is readily apparent that marked differences exist in the number of features that distinguish between COD when tallying results across platforms. However we caution the reader that the purpose of this experimental approach was primarily the qualitative evaluation of distinguishing differences in transcript, protein, and/or metabolite expression between COD. While the next generation of experiments would be well served to use seeds matched for physiological age across all omics platforms, these initial experiments were performed using available mature seed with the exception of the initial work on transcript expression which required embryos to be harvested in the field and immediately frozen in liquid nitrogen.

Many factors other than the biology of the system could account, at least in part, for the numeric differences observed. For example, the use of the soy-array to identify differentially expressed transcripts or the use of only one of an almost endless number of assay conditions that have been developed for proteomic or metabolomic analyses are likely to make the numeric differences noted unreliable. Nonetheless, the magnitude of the observed differences indicates the potential merit of these approaches as a fertile source of information for plant breeders.

Overall, the 'omics' chosen for a particular set of experiments should depend on the question being asked. While considerable information can be gleaned from each 'platform, the biological activity assessed is interactive, and accordingly, the greatest insights are likely to emerge through the integration of complimentary data sets. Transcriptomics, proteomics, and metabolomics are just three of these [28].

## Conclusions

We have shown that two genetically diverse dry bean germplasm can be easily differentiated using a suite of three 'tools (transcriptomics, proteomics, and metabolomics). Using this panel of techniques we have provided a glimpse into a signature which can be used to determine COD. Furthermore, known genetic distances among cultivars and between COD were validated using proteomics and metabolomics. 'Omic' signatures unique to *P. vulgaris *germplasm may be useful to assess complex traits or reactions to biotic and abiotic stress and to incorporate genetic diversity in breeding efforts. This work compliments other techniques currently employed by breeders to assess dry bean genetic diversity. As signatures are further developed in future work, they have the potential to guide those engaged in crop improvement for selecting traits of agronomic and nutritional importance.

## Methods

### Bean Material

Navy bean (cv. Seahawk, cv. Norstar, and cv. Vista) and white kidney bean (cv. Beluga, cv. Silver Cloud, and cv. Lassen) cultivars representative of the two market classes were selected for this study to represent the Middle American and Andean COD, respectively. Seeds from each of the market classes used in this study were obtained from field grown plants at the Colorado State University Agricultural Research, Development and Education Center (ARDEC), Fort Collins, CO. The bean seed used for analysis within each omics approach was of the same physiological age but differed across omics platforms. Seeds for transcriptomics analyses were collected from plants at two and three weeks after anthesis (plant flowering), immediately frozen in liquid nitrogen and stored at -80°C. Seeds for proteomics and metabolomics were harvested at full maturity and air dried and stored at room temperature (RT, 22 ± 2°C) until use.

### RNA isolation

RNA was extracted from navy and white kidney bean seeds from field grown plants in 2007. Seeds were collected in the field and immediately frozen as described above. Two cultivars, Silver Cloud (white kidney) and Norstar (navy) were selected for spotted array gene expression analysis. After storage, frozen seeds were separated from pods and RNA was isolated according to the TRIzol reagent protocol (Life Technologies, Gaithersburg, MD). The RNA sample was purified with the RNeasy Mini Kit (Qiagen, Valencia, CA) according to Affymetrix (Santa Clara, CA) instructions. RNA concentrations and purity were determined using an ND-1000 NanoDrop spectrophotometer (Thermo Scientific NanoDrop, Wilmington, DE). RNA integrity was evaluated by the Experion Bioanalyzer Automated Electrophoresis System (Bio-Rad Laboratories, Hercules, CA).

### Microarray hybridization

Since a commercial *Phaseolus vulgaris *microarray was not available, a soybean (*Glycine max *L. Merr.) array was selected because it was the closest phylogenetic relative among available arrays [29]. Although *Glycine max *and *Phaseolus vulgaris *differ in chromosome number and genome size (the soybean genome is twice as large as common dry bean), linkage mapping of DNA markers found an average conserved block length of 13.9 cM between the two genomes indicating high conversation and preservation [29,30]. Twenty-five μg of RNA was used to prepare complimentary DNA for spotted 2-color microarray analysis. Complimentary DNA (cDNA) was synthesized using the Superscript III kit (Invitrogen, Carlsbad, CA) and hybridized to a microarray developed for soybean [23] using the Genisphere Array 50 kit (Genisphere, Hatfield, PA). Briefly, RNA (29.5 μL) was mixed with dye-appropriate RT primer (1.5 μL) and heated to 80°C for 10 min, and transferred to ice for 2 min. Superase-in RNase inhibitor (1 μL) was added before 18 μL of reaction mix composed of 5× Superscript III first strand buffer (10 μL), 10 mM dNTP mix (2 μL), 0.1 M dithiotreitol (4 μL) and Superscript III enzyme (4 μL). After incubation at RT for 5 min, tubes were placed in an iQ iCycler (Bio-Rad) for 2 h at 50°C. The reaction was stopped with 0.5 M NaOH/50 mM EDTA (7 μL) followed by incubation at 65°C for 10 min and neutralized with 1 M Tris-HCl (10 μL). cDNA from the navy and white kidney bean samples were combined and purified using Geneclean Turbo kit (Qbiogene, Carlsbad, CA). The purified cDNA was stored at -20°C until use. Arrays were cross-linked by exposure to 65 mJ of UV irradiation and blocked by incubation in 250 mL prehybridization solution [5× SSC (18% Sodium Chloride, 9% Sodium Citrate) buffer, 0.1% SDS and 0.01% BSA solution] at 42°C for 1-2 h. The arrays were dried by centrifugation at RT for 5 min and placed into hybridization chambers and incubated at 42°C for 15 min. Formamide-based buffer (2×, 50 μL) and LNA dT blocker (2 μL) were added to the cDNA sample and heated for 10 min at 80°C. A cover slip was placed on the array and the cDNA mix was introduced between the cover slip and the array surface. The solution was distributed evenly on the array by capillary action. Water (15 μL) was added to the wells of the array chambers followed by incubation for 16 h at 42°C. The array was vigorously washed 3 times for 2 min, first with 2× SSC and 0.2% SDS (250 mL) followed by 2× SSC (250 mL) and then with 0.2× SSC (250 mL). The arrays were centrifuged for 3 min (1000 × g) to dry and warmed at 42°C for 15 min. A mix of 2× formamide-based buffer (42 μL), water (35 μL) and 3.5 μL each of Cy3 and Cy5 was prepared in the dark and incubated at 80°C for 10 min. The hybridization mix was then injected onto the soybean cDNA microarray. Arrays were incubated at 42°C for 3 h. After the second hybridization step, the arrays were washed and dried as described above and immediately scanned with a Genepix 4000B scanner (Molecular Devices, Sunnyvale, CA).

Scanned images were analyzed using GenePix Pro 6.0 software (Molecular Devices) where spot features were aligned for annotation. Poorly hybridized spots were discarded from analysis using the software parameters and the intensities of each dye were quantified. Fluorescence intensities were normalized and exported for statistical analyses.

### Protein isolation and two-dimensional gel electrophoresis (2DGE)

2DGE was performed with bean extracts according to previously published methods [31]. Approximately 15 mg of bean seed samples from each of the six cultivars listed above were suspended in sample lysis buffer, containing 7 M urea, 2 M thiourea, 40 mM DTT, 2% CHAPS, 1% Pharmalyte pH 3-10 (GE Healthcare, Piscataway, NJ), and trace amount of bromophenol blue (BPB). Insoluble debris was removed after centrifugation for 30 min (1000 × g, 15°C). The supernatant was recovered and used for analysis. Protein concentration was quantified using the Bradford assay.

Bean seed protein (200 μg) was applied to reswelled, immobilized pH gradient (IPG) strips (pH 4-7L, 24 cm; GE Healthcare). The isoelectrofocusing conditions were as follows: 10V to 300 V for 3 h and at 5000 V for a total of 95 kVh at 20°C. After isoelectrofocusing, the strips were equilibrated in a buffer containing 30% glycerol, 6 M urea, 2% SDS, 10 mg/mL dithiothreitol (DTT) and 0.05 M Tris-HCl (pH 6.8) for 15 min and then for an additional 15 min in equilibration buffer in which 42.5 mg/mL iodoacetamide replaced the DTT. The strips were positioned at the top of 13-16% gradient polyacrylamide gels with 0.5% agarose containing Laemmli sample buffer [31]. SDS-PAGE was performed in Laemmli electrophoresis buffer at 150 V at RT. Proteins were stained with alkaline ammoniacal silver staining [32] scanned with a GS-800 Calibrated Densitometer (Bio-Rad) and analyzed using PDQuest v7.1.1 software (Bio-Rad).

### Analysis of gels

Samples were analyzed using 2DGE to obtain quantitative protein profiles within the molecular weight range of 25 to 150 kDa. Each bean cultivar was run in duplicate. Protein spots were automatically detected using PDQuest v7.1.1 software. All spots were also manually confirmed. Images of duplicate gels were superimposed and a master gel generated for each of the six cultivars. Proteins were quantified using spot densitometry.

Comparisons of the 2DGE protein patterns generated an inclusion list for only those proteins that differed significantly between the two COD (greater than 2-fold change). Protein patterns were also compared between cultivars within each COD. Although out of the scope of this work, these spots can be used in the future for protein identification using MALDI TOF MS.

### Metabolite extraction and analysis

It is imperative that the majority of the proteins within the dry bean samples are removed prior to metabolomic fingerprinting in order to avoid confounded results from possible protein fragments. The dried bean seeds were boiled to denature proteins and freeze dried for storage. The proteins were then precipitated during the metabolite extraction process using cold ethanol. The boiling procedure is as follows: 0.5 kg of bean was soaked in distilled water for 3 h at RT. After 3 h the beans were drained, rinsed thoroughly with deionized water, and blanched for 5 min at 93°C. Beans were boiled for 60 min in a 1.5% KCl solution using a pressure cooker. Finally, beans were freeze dried (Genesis SQ25LL, Virtis Company, Gardiner, NY) to powder form and stored at -80°C until use.

Metabolites were extracted using cold ethanol (65%, -20°C). Approximately 2.5 g of freeze dried bean powder was added to ethanol (65%, 50 mL) and vortexed to ensure complete mixing. The mixture was sonicated at RT for 2 h and centrifuged (1000 × g, 10 min) to separate the insoluble material from the ethanol extract. The extract was decanted into a clean conical tube and stored at -20°C until analysis up to 1 mo.

### Ultra Performance Liquid chromatography-Mass Spectrometry (UPLC-MS)

Sample separation was performed using an Acquity UPLC under the control of MassLynx software (Waters, Millford, MA, USA). The sample set was randomized and held in an 8°C sample manager during the analysis. For each chromatographic run, a 1 μL sample injection was loaded to a 1.0 × 100 mm Waters Acquity UPLC BEH C18 column with 1.7 μm particle size held at 40°C. Separation was performed by reverse phase chromatography at a flow rate of 0.15 ml/min. The eluent consisted of water and methanol (Fisher, Optima^® ^LC/MS grade) supplemented with formic acid (Fluka, LC/MS grade) in the following proportions: Solvent A = 95:5 water:methanol + 0.1% formic acid; Solvent B = 5:95 water:methanol + 0.1% formic acid. The separation method is described as follows: 0.1 min hold at 100% A, 14.9 min linear gradient to 100% B, 5 min hold at 100% B, 1 min linear gradient to 100% A, and 1 min hold at 100% A. A blank injection of water and 15 min chromatographic run was preformed between samples to eliminate possible carryover of analytes and to re-equilibrate the column. This cleaning method is described as follows: 0.1 min hold at 100% A, 2.9 min linear gradient to 100% B, 1 min hold at 100% B, 3 min linear gradient to 100% A, and 8 min hold at 100% A for equilibration. The flow rate for all steps was held at 0.15 ml/min.

Eluate was directed to a Q-TOF Micro quadrupole orthogonal acceleration time-of-flight mass spectrometer controlled with MassLynx software (Waters/MicroMass, Millford, MA, USA) using electrospray ionization in the positive mode (ESI+). Mass data were collected between 50 and 1000 m/z at a rate of two scans per second with a 0.1 second interscan delay. The voltage and temperature parameters were tuned for general profiling as follows: capillary = 3000 V; sample cone = 30 V; extraction cone = 2.0 V; desolvation temperature = 300°C; and source temperature = 130°C. Mass spectral peaks were centered during acquisition producing centroid data. Leucine Enkephalin was infused via an orthogonal ESI probe and baffle system (LockMass) which allowed reference ions to be detected for a single half-second scan every 10 s in an independent data collection channel. The standard mass was averaged across 10 scans providing a continuous reference for mass correction of analyte data.

Chromatographic and spectral LC-MS peaks were detected, extracted, and aligned using MarkerLynx software (Waters, Millford, MA, USA). Chromatographic peaks were detected between 0 and 18 min with a retention time error window of 0.1 min. Apex track peak detection parameters were used, automatically detecting peak width and baseline noise. No smoothing was applied. To reduce the detection and inclusion of noise as data, an intensity threshold value of 40 and a noise elimination value of 6 were used. Mass spectral peaks were detected between 50 and 1000 m/z with a mass error window of 0.07 m/z. The de-isotoping function was enabled to eliminate the inclusion of isotopic peaks. A matrix of features as defined by retention time and mass was generated, and the relative intensity of all features, as determined by area, was calculated for all individual samples. Potential effects of technical variability were minimized by normalizing the intensity values to the total ion current (TIC) such that the summation of all feature intensities in each individual sample were equal.

### Data analysis

For microarray analysis, data were imported into Partek Discovery Suite software (Partek, St. Louis, MO), PC and 1-way ANOVA (random effects) (p < 0.01) analyses were performed. For 2DGE, PC, hierarchal clustering based on Euclidian distances, and 1-way ANOVA (random effects) (p < 0.05) analyses were carried out using Partek Discovery Suite software. Finally, the LC-MS feature matrix was mean centered and imported into SIMCA-P+ software (Umetrics, Inc., Umeå, Sweeden). PC analysis was performed using Pareto scaling. Hierarchal clustering based on Euclidian distance and 1-way ANOVA (random effects) (p < 0.05) analyses were performed using Partek Discovery Suite software. Fold change for both proteomics and metabolomics was calculated using Partek Discovery Suite.

## Authors' contributions

MM carried out the metabolomics studies, statistical analysis associated with metabolomics analysis, analysis of proteomics data, and drafted the manuscript. VF carried out the transcriptomics studies, the statistics associated with the transcriptomics data and helped draft the manuscript. ML carried out the LC-MS work presented here, assisted MM with metabolomics analysis and helped edit and draft the manuscript. ER participated in the study design and helped draft the manuscript. HT participated in the study design, assisted with the statistical analysis and helped to draft the manuscript. MB participated in the study design and helped to draft the manuscript. All authors read and approved the final manuscript.

## References

[B1] Gepts P Aragao FL deBarros E Blair MW Brondani R Broughton W Galasso I Hernandez G Kami J Lariguet P Moore PH, Ming R Genomics of Phaseolus Beans, a Major Source of Dietary Protein and Micronutrients in the Tropics Genomics of Tropical Crop Plants 2008 Philadelphia: Springer 113 143 full_text

[B2] Kaplan L Kaplan LN Gepts P Dordrecht Phaseolus in archaeology Genetic Resources of Phaseolus Beans 1988 Netherlands: Kluwer Academic Publishers 125 142

[B3] Gepts P Debouck DG Voysest O, Van Schoonhoven A Oxon Origin, domestication, and evolution of the common bean, Phaseolus vulgaris Common Beans: Research for Crop Improvement 1991 UK: CAB International 7 53

[B4] Kaplan L Archeology and domestication in American Phaseolus (beans) Economic Botany 1965 19 358 368

[B5] Kaplan L McNeish RS Prehistoric bean remains from caves in the Ocampo region of Tamaulipas, Mexico 1960 19 33 35

[B6] Kaplan L Lynch TF Phaseolus (Fabaceae) in archaeology: AMS radiocarbon dates and their significance for pre-Colombian agriculture Economic Botany 1999 53 3 261 272

[B7] Piperno DR Dillehay TD Starch grains on human teeth reveal early broad crop diet in northern Peru Proceedings of the National Academy of Sciences of the United States of America 2008 105 50 19622 19627 10.1073/pnas.0808752105 19066222 PMC2604935

[B8] Koinange EMK Singh SP Gepts P Genetic control of the domestication syndrome in common bean Crop Science 1996 36 4 1037 1045 10.2135/cropsci1996.0011183X003600040037x

[B9] Singh SP Gepts P Debouck DG Races of Common Bean (Phaseolus-Vulgaris, Fabaceae) Economic Botany 1991 45 3 379 396

[B10] Kwak M Kami JA Gepts P The Putative Mesoamerican Domestication Center of Phaseolus vulgaris Is Located in the Lerma-Santiago Basin of Mexico Crop Science 2009 49 2 554 563 10.2135/cropsci2008.07.0421

[B11] Singh SP Nodari R Gepts P Genetic Diversity in Cultivated Common Bean .1. Allozymes Crop Science 1991 31 1 19 23 10.2135/cropsci1991.0011183X003100010004x

[B12] Gepts P Bliss FA Phaseolin Variability Among Wild and Cultivated Common Beans (Phaseolus-Vulgaris) from Colombia Economic Botany 1986 40 4 469 478

[B13] Gepts P Kmiecik K Pereira P Bliss FA Dissemination Pathways of Common Bean (Phaseolus-Vulgaris, Fabaceae) Deduced from Phaseolin Electrophoretic Variability .1. the Americas Economic Botany 1988 42 1 73 85

[B14] Koenig R Gepts P Allozyme Diversity in Wild Phaseolus-Vulgaris - Further Evidence for 2 Major Centers of Genetic Diversity Theoretical and Applied Genetics 1989 78 6 809 817 10.1007/BF00266663 24226011

[B15] Freyre R Rios R Guzman L Debouck DG Gepts P Ecogeographic distribution of Phaseolus spp (Fabaceae) in Bolivia Economic Botany 1996 50 2 195 215

[B16] Velasquez VLB Gepts P Rflp Diversity of Common Bean (Phaseolus-Vulgaris) in Its Centers of Origin Genome 1994 37 2 256 263 10.1139/g94-036 18470075

[B17] Kami J Velasquez VB Debouck DG Gepts P Identification of Presumed Ancestral Dna-Sequences of Phaseolin in Phaseolus-Vulgaris Proceedings of the National Academy of Sciences of the United States of America 1995 92 4 1101 1104 10.1073/pnas.92.4.1101 7862642 PMC42645

[B18] Chacon SMI Pickersgill B Debouck DG Arias JS Phylogeographic analysis of the chloroplast DNA variation in wild common bean (Phaseolus vulgaris L.) in the Americas Plant Systematics and Evolution 2007 266 3 175 195 10.1007/s00606-007-0536-z

[B19] Blair MW Giraldo MC Buendia HF Tovar E Duque MC Beebe SE Microsatellite marker diversity in common bean (Phaseolus vulgaris L.) Theoretical and Applied Genetics 2006 113 1 100 109 10.1007/s00122-006-0276-4 16614831

[B20] Blair MW Pedraza F Buendia HF Gaitan-Solis E Beebe SE Gepts P Tohme J Development of a genome-wide anchored microsatellite map for common bean (Phaseolus vulgaris L.) Theoretical and Applied Genetics 2003 107 8 1362 1374 10.1007/s00122-003-1398-6 14504741

[B21] Yang SS Valdes-Lopez O Xu WW Bucciarelli B Gronwald JW Hernandez G Vance CP Transcript profiling of common bean (Phaseolus vulgaris L.) using the GeneChip Soybean Genome Array: optimizing analysis by masking biased probes BMC Plant Biol 2010 10 85 10.1186/1471-2229-10-85 20459672 PMC3017814

[B22] Bar-Or C Czosnek H Koltai H Cross-species microarray hybridizations: a developing tool for studying species diversity Trends Genet 2007 23 4 200 207 10.1016/j.tig.2007.02.003 17313995

[B23] Vodkin LO Khanna A Shealy R Clough SJ Gonzalez DO Philip R Zabala G Thibaud-Nissen F Sidarous M Stromvik MV Microarrays for global expression constructed with a low redundancy set of 27,500 sequenced cDNAs representing an array of developmental stages and physiological conditions of the soybean plant BMCGenomics 2004 5 1 73 10.1186/1471-2164-5-73 PMC526184 15453914

[B24] Wink M Plant secondary metabolism: Diversity, function and its evolution Nat Prod Commun 2008 3 8 1205 1216

[B25] McClean P Lavin M Gepts P Jackson S Stacey G Phaseolus vulgaris: A Diploid Model for Soybean Genetics and Genomics of Soybean 2008 New York: Springer 55 76 full_text

[B26] Common Bean Coordinated Agricultural Project http://www.reeis.usda.gov/web/crisprojectpages/219849.html

[B27] Subramanian A Tamayo P Mootha VK Mukherjee S Ebert BL Gillette MA Paulovich A Pomeroy SL Golub TR Lander ES Gene set enrichment analysis: A knowledge-based approach for interpreting genome-wide expression profiles Proceedings of the National Academy of Sciences of the United States of America 2005 102 43 15545 15550 10.1073/pnas.0506580102 16199517 PMC1239896

[B28] Joyce AR Palsson BO The model organism as a system: integrating 'omics' data sets NatRevMolCell Biol 2006 7 3 198 210 10.1038/nrm1857 16496022

[B29] Choi HK Mun JH Kim DJ Zhu H Baek JM Mudge J Roe B Ellis N Doyle J Kiss GB Estimating genome conservation between crop and model legume species ProcNatlAcadSciUSA 2004 101 43 15289 15294 10.1073/pnas.0402251101 PMC524433 15489274

[B30] Boutin SR Young ND Olson TC Yu ZH Vallejos CE Shoemaker RC Genome conservation among three legume genera detected with DNA markers Genome 1995 38 5 928 937 10.1139/g95-122 18470218

[B31] Lee SG Mykles DL Proteomics and signal transduction in the crustacean molting gland Integrative and Comparative Biology 2006 46 6 965 977 10.1093/icb/icl047 21672800

[B32] Oakley BR Kirsch DR Morris NR A Simplified Ultrasensitive Silver Stain for Detecting Proteins in Polyacrylamide Gels Analytical Biochemistry 1980 105 2 361 363 10.1016/0003-2697(80)90470-4 6161559

